# Interrupting Malaria Transmission: Quantifying the Impact of Interventions in Regions of Low to Moderate Transmission

**DOI:** 10.1371/journal.pone.0015149

**Published:** 2010-12-02

**Authors:** Michelle L. Gatton, Qin Cheng

**Affiliations:** 1 Malaria Drug Resistance and Chemotherapy Laboratory, Queensland Institute of Medical Research, Brisbane, Queensland, Australia; 2 Griffith Medical Research College, Brisbane, Queensland, Australia; 3 Drug Resistance and Diagnostics, Australian Army Malaria Institute, Brisbane, Queensland, Australia; Université Pierre et Marie Curie, France

## Abstract

Malaria has been eliminated from over 40 countries with an additional 39 currently planning for, or committed to, elimination. Information on the likely impact of available interventions, and the required time, is urgently needed to help plan resource allocation. Mathematical modelling has been used to investigate the impact of various interventions; the strength of the conclusions is boosted when several models with differing formulation produce similar data. Here we predict by using an individual-based stochastic simulation model of seasonal *Plasmodium falciparum* transmission that transmission can be interrupted and parasite reintroductions controlled in villages of 1,000 individuals where the entomological inoculation rate is <7 infectious bites per person per year using chemotherapy and bed net strategies. Above this transmission intensity bed nets and symptomatic treatment alone were not sufficient to interrupt transmission and control the importation of malaria for at least 150 days. Our model results suggest that 1) stochastic events impact the likelihood of successfully interrupting transmission with large variability in the times required, 2) the relative reduction in morbidity caused by the interventions were age-group specific, changing over time, and 3) the post-intervention changes in morbidity were larger than the corresponding impact on transmission. These results generally agree with the conclusions from previously published models. However the model also predicted changes in parasite population structure as a result of improved treatment of symptomatic individuals; the survival probability of introduced parasites reduced leading to an increase in the prevalence of sub-patent infections in semi-immune individuals. This novel finding requires further investigation in the field because, if confirmed, such a change would have a negative impact on attempts to eliminate the disease from areas of moderate transmission.

## Introduction

Past malaria elimination campaigns have resulted in more than 40 countries becoming malaria-free [1], [2], reinforcing that local elimination is possible, particularly from marginal areas with unstable transmission where an estimated 1 billion people live [3]. After two decades focused on malaria control [4], elimination is back in the spotlight with 39 countries planning for, or already committed to, elimination [5]. Malaria elimination entails the interruption of malaria transmission to zero and then the maintenance of this state by controlling the importation of infections. While the WHO malaria elimination guidelines provide valuable information on how to transition from control to elimination, they do not address the likely impact of the suggested interventions or the time frame required to achieve elimination in different transmission settings [6].

Mathematical models have long been used to model disease patterns with the main practical aim of understanding the dynamics of transmission and impact of interventions well enough to guide and manage control programs [7]. To this end, numerous models have been developed and used to estimate the impact of interventions including, but not limited to, insecticide treated nets (ITNS) [8], [9], [10], artemisinin combination therapy (ACT) [11], case management [12] and vaccines [10], [13] in specific settings, usually Africa. The potential for elimination has also been investigated using more generic frameworks [14], [15]. In an ideal world countries would deploy an integrated malaria control program using a combination of interventions which target different aspects of transmission, to increase the chances of success [16]. The financial and logistic reality is that most regions need to select a subset of interventions and therefore need information about the relative effects of different combinations.

In this study we use an individual-based discrete-time stochastic simulation model of seasonal *Plasmodium falciparum* transmission to compare the impact of selected interventions in low to moderate transmission settings. The interventions included in the study focus on chemotherapy and reduction of human-mosquito contact through use of untreated bed nets. In general, the results support those from previous models, but also provide novel predictions of long-term impacts on parasite carriage and population structure when treatment coverage is improved.

## Materials and Methods

**Figure 1:**
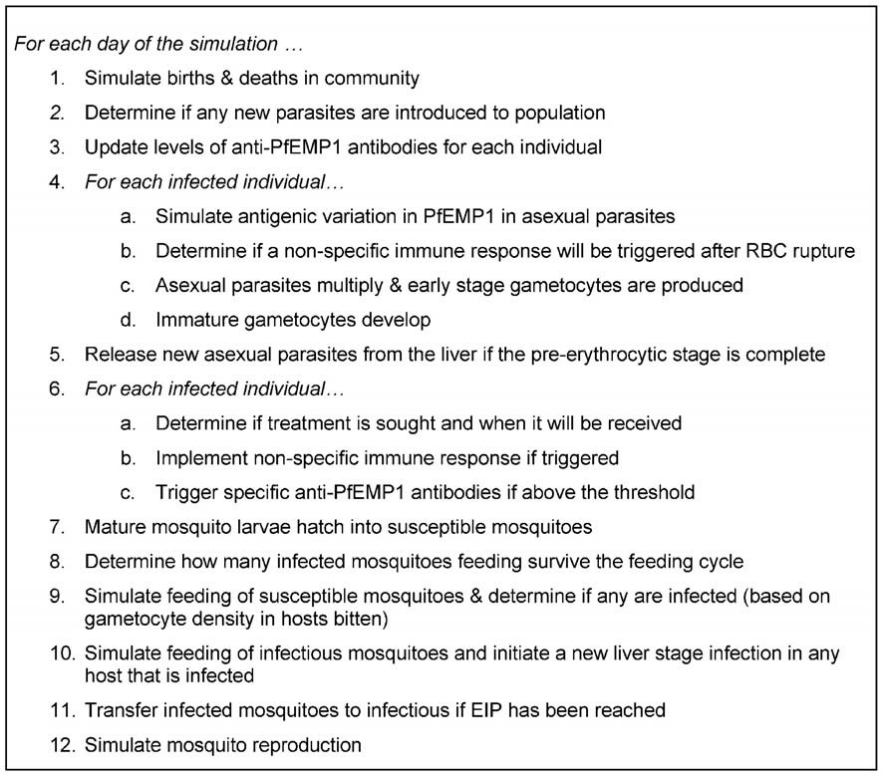
Description of processes, and order of application, for the malaria transmission model.

The model expands an existing parameterized within-host dynamics model, which was validated using data from malaria naïve patients [17], [18] to encompass a population of individuals and parasites linked by mosquito transmission. This model is built on the premise that the biology of the parasite and hosts, and their interactions, is independent of geographic location. The epidemiology of the disease is defined by the combination of social and behavioural characteristics within the human population, the mosquito population and climatic variables. These factors influence the exposure of individuals to the parasite, resulting in each host mounting an immune response dependent on their exposure. A simple description of the processes incorporated into the model, and the order of application, are outlined in Figure 1. Parameter values are given in Table 1.

**Table 1:** Description of variables used in the model.

Variable/Description	Data	Value used
Probability of new parasite being introduced to population	-	0.02/day
Probability of mosquito surviving one feeding cycle	0.58–0.70 [14], [43]	0.65
Length of feeding cycle	2.7–3.7 days [14], [43]	3 days
Probability of mosquito feeding on human	0.72–0.95 [14]	0.75
Size of mosquito blood meal	0.97 µl (median value) [44]	1 µl
Length of extrinsic incubation period (EIP)	Temp. dependent [45]	9 days (at 28°C)
Larval development time	Temp. dependent [45]	12 days (at 28°C)
Probability of infectious mosquito bite resulting in liver stage infection	0.583 [29]	0.6
Duration of liver stage infection	6 days [46]	6 days
Proportion of gametocytes that are male	0.2–0.3 [47], [48]	0.25
Total number of antigenically distinct *var* genes in parasite population	350–1230* [49]	400
Number of *var* genes per parasite	59 [50]	60
Maximum parasite replication rate in the absence of any immune response (*R*)	8–32 [51]	16

*represents number of sequence types which may be more than antigenic types.

### Human population dynamics and disease

The initial age distribution and death-rates in a community of 1,000 people were based on those reported for Gabon [19], [20]. To maintain a closed population, a birth is assumed whenever a death occurred. The new individual is naïve to malaria with no protective maternal antibodies.

Within an individual host, three immune responses are simulated: 1) a non-specific immune (NSI) response based on the total number of rupturing parasites, 2) a non-variant immune response specific to individual parasite clones, and 3) a PfEMP1 variant-specific antibody response. The mathematical form of these responses, along with a sensitivity analysis, was first reported in Paget-McNicol et al. [21], with some subsequent refinements [17], [18]. The majority of the parameters in the model were fitted using data from patients infected with the El Limon and Santee Cooper strains of *P. falciparum* [17]. The triggering and dynamics of anti-PfEMP1 antibodies to specific PfEMP1 variants are as previously defined [18]. The magnitude of the NSI response, referred to here as fever (*F_t_*,), has the mathematical form
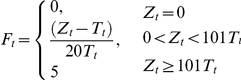
where *Z_t_* is the number of infected RBC replicating on day *t* and *T_t_* is the fever threshold as previously defined [18]. Previous model fitting was based on *F_t_* values >2 corresponding to fevers >104°F [17]. A lower value of 0.3 is used here as a threshold for when the NSI response was sufficient to cause clinical symptoms. A symptomatic episode is defined as one or more fever events not separated by more than 7 days.

### Parasite dynamics

The *var* genes (which encode PfEMP1) allocated to each parasite clone are 1) randomly selected (with replacement) from the global population of variants for introduced parasites, or 2) selected from those present in the male and female gametes during recombination in the mosquito. No within gene recombination is included in the model. The calculations for parasite switching and the switching probabilities for each antigenically distinct *var* gene in the population (*p_j_*) were as previously reported assuming a global population of 400 antigenically distinct genes [18]. It is important to note that the number of antigenically distinct *var* genes is less than the number of unique *var* gene sequences due to antigenic cross-reactivity. This point is most clearly highlighted by the parity-dependent antibody response which interferes with binding of parasites to the placenta, even though the PfEMP1 variants thought to be responsible for the binding are structurally similar but unique in sequence [22]. Little, if any data exists on the number of antigenically distinct PfEMP1 variants circulating within populations, so the value of 400 is assumed.

Introductions of new parasite types into the population are permitted with a daily probability of 0.02 and implemented by establishing a liver-stage infection with the new parasite in a randomly selected host.

Asexual parasites replicate every 2 days and early stage gametocytes are produced. The replication and implementation of the non-variant specific immunity uses the mathematical form previously described [18]:
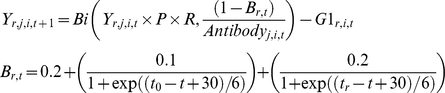
where *Y_r,j,i,t_* is the number of clone *r* asexual parasites expressing PfEMP1 variant *i* in host *j* on day *t*, *P* is a conversion factor from number of parasites per µl of blood to total number of parasites in a host (assumed to be 5×10^6^), *R* is the maximum replication rate of parasites in the absence of any immune response, *Antibody_j,i,t_* is the amount of anti-PfEMP1 antibody *i* for host *j* on day *t*, *t_r_* is the first day of infection with parasite *r* and *t_0_* is the first day of the first infection within the host, and 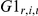 are the number of early stage gametocytes of parasite type *r* that express PfEMP1 variant *i* produced on day *t*. It is assumed that the conversion rate of asexual parasites to G1 gametocytes follows a log-normal distribution with 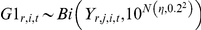 and η = -1.6 when *F_t_* = 0, or -1.4 when *F_t_*>0. These conversion rates are higher than those reported for the conversion from asexual to circulating mature gametocytes [23] to account for death of early stage gametocytes due to susceptibility to the NSI response and circulating anti-PfEMP1 antibodies [24], [25].

Gametocyte dynamics are as previously described [26]. When gametocytes from more than one parasite type are ingested by the mosquito, a male and female are randomly selected to mate based on the relative density of each parasite.

All asexual parasites and gametocytes are susceptible to the host NSI response [24], [27]. The number of parasites surviving an active NSI response (*F_t_* >0) follows a binomial distribution with survival probability exp(−α×*F_t_*) and α = 1, 2, 1/2.75 and 1/1.5 for ring stage asexual parasites, late stage asexual parasites, early stage gametocytes and mature gametocytes, respectively. The scaling factor, α, was used to alter the susceptibility of parasites based on their life-stage. The difference in survival of late stage asexual parasites compared to ring-stage parasites reflects experimental data [27], while the relative gametocyte susceptibility assumes that gametocytes, like stress-induced stages in other organisms, are more resilient to increased temperature than asexual parasites. In addition, it is assumed that sequestration of early stage gametocytes in the spleen and bone marrow provides some protection, relative to mature gametocytes, from the NSI effect.

### Mosquito infection and transmission

The mosquito population consists of susceptible, infected (not infectious) and infectious mosquitoes. Super-infection is not permitted and mosquitoes do not recover from infection. The baseline number of mosquitoes (*mos_0_*) in the population is specified, as well as an amplification factor, *a_p_*, for each day of the year. The amplification factor represents the relative number of mosquitoes born and is used to introduce seasonality. The seasonal pattern was selected so that ∼95% of mosquito exposure occurred in 7 months of each year, a pattern similar to that reported for Sotuba, Mali [28]. The number of new mosquitoes emerging to join the susceptible population on day (*t+12*), *mb_t+12_*, is 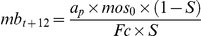, where *S* is the probability of a mosquito surviving one feeding cycle and *Fc* is the length of the feeding cycle.

It is assumed each mosquito feeds randomly once per feeding cycle, with the day of feeding within the cycle randomly assigned and fixed for the life of the mosquito. Mosquito survival is determined once per feeding cycle using a binomial distribution with probability *S*. Infection of a susceptible mosquito during feeding is dependent on at least one male and one female gametocyte being ingested in the bloodmeal. The probability of an infectious mosquito bite generating a new liver-stage infection in the host is fixed at 0.6 [29].

### Simulations

The model was compiled and executed using the Griffith University Research Computing Services Sun Fire V20Z HPC Cluster. Each simulation considered a village of 1,000 individuals and used the same host, parasite and entomological parameter values. Different transmission settings were achieved by altering *mos_0_* and each was run for 10,000 days to establish baseline transmission and host immunity profile. The community profile on day 10,000 was used as a common start point for simulations incorporating malaria interventions.

Three primary interventions (improved treatment rates of symptomatic individuals (T90), mass drug administration (MDA), and untreated bed nets), plus combinations, were simulated for 5,000 days, with 10–20 simulations for each transmission/intervention combination. Model output was summarised, graphed and analysed using Sigma Plot (Version 11) and SAS for Windows (Version 5.1.2600, SAS Institute Inc). Each intervention was assessed for its ability to interrupt malaria transmission for at least 150 days, that is, there are no infectious mosquitoes in the population for at least 150 days. We have classified parasite introductions (importations) as being controlled if they do not become established in the population by surviving between transmission seasons.

#### Baseline conditions

Each symptomatic episode had a 50% chance of receiving treatment. It is recognised that often there is a delay between becoming symptomatic and seeking treatment. In the baseline simulation it is assumed that 10% of the people who seek treatment get that treatment within 24 hours of becoming symptomatic, while 10%, 10%, 25%, 25% and 20% will receive treatment 2, 3, 4, 5 and 6 days after becoming symptomatic, respectively. This distribution of when treatment was received following the onset of fever was based on a variety of data on treatment seeking behaviour in Vietnam and Africa [30], [31], [32], [33], [34]. In all simulations the use of anti-malarial drugs to treat non-malarious fevers occurred randomly at a rate of 0.1% of the population per day.

It is assumed that only one long-acting drug or drug combination is administered, and that it is 100% effective with no resistance. 100% of parasites are killed for the first 30 days post-treatment, followed by a rapid decline in effectiveness so that by day 35 any residual drug is ineffective, in line with elimination profile of sulfadoxine-pyrimethamine.

#### T90 intervention

Each symptomatic episode had a 90% chance of receiving treatment with 20%, 40%, 30%, 5%, 3%, 2% of these people receiving treatment 1, 2, 3, 4, 5 and 6 days after becoming symptomatic, respectively. The shortening of the time to treatment is representative of the changes seen after provision of health posts within communities in Vietnam [30].

#### Bed nets

80% untreated bed net coverage is assumed with each net providing protection from 80% of mosquito bites. Nets are randomly assigned to individuals at the start of the intervention and it is assumed that the bed nets are used every day. The baseline treatment conditions are maintained.

The nets are untreated so only act to reduce human-mosquito contact, having no effect on survival of mosquitoes. Hence the impact of the intervention will be conservative compared to the long-lasting ITNs currently being deployed. Inclusion of insecticide effects is beyond the scope of the current work due to the need to better quantify important ecological parameters such as the killing probability, possible repellancy effects and mosquito behavioural changes [35].

Where a feeding mosquito fails to get a blood meal due to a bed net, the model assumes the mosquito to seek another host, up to a maximum of three, after which it will source a blood meal from a non-human source. The probability of survival during this process is assumed to remain unchanged.

#### MDA

At the start of the intervention 100% of the population received a curative dose of the same anti-malarial drug used to treat symptomatic individuals. The treatment is repeated again 40 days into the intervention, after which no further actions are taken other than the initial baseline conditions.

### Sensitivity analysis for bed net and symptomatic treatment interventions

The sensitivity of the model output to coverage levels for the bed net intervention and treatment of symptomatic cases was assessed by comparing the cumulative entomological inoculation rate (EIR) and fever during the first five years following implementation. For the bed net intervention, additional sensitivity analysis was performed in which the assumed reduction in mosquito exposure cause by a bed net was varied between 75 and 85%.

## Results

The model was used to simulate infection and disease in low to moderate transmission settings with EIRs between 2 and 15 infectious bites per person per year. Under baseline conditions (prior to interventions) with an EIR of ∼3 (low transmission), 12–15% of the population had patent parasitemia at the peak of the transmission season with <1% of individuals having multiple infections. The median prevalence of patent parasitemia (≥100 parasites/µl) in the population varied from 1.9 to 2.6% across the simulations, comparable to the median for children aged 4 to 8 years (2.0–2.9%). The mean number of fever episodes in the 0–3 year age group was 1.29–1.59 per person per year, varying between simulations, being 84–90% of the actual number of infections received (1.49–1.87 per person per year).

Baseline simulations in the moderate transmission scenario (EIR ∼15) represent a community where the frequency of fever episodes decreased with age from 3.5–3.9 per person per year in the 0–3 year age group to 1.7–2.4 fevers per person per year in >14 year olds. During the peak transmission season up to 35% of the population had patent parasitemia with 8–10% infected with ≥2 parasite strains. The median prevalence rate over the simulation period was 5.3–7.9% in the entire population and 7.5–9.2% in children aged 4–8 years, varying between individual simulations.

**Figure 2:**
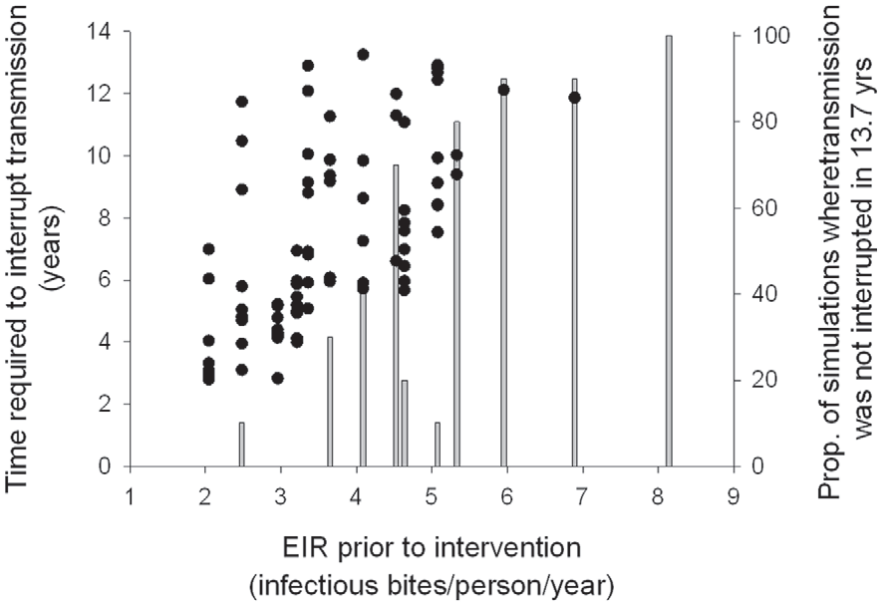
Times required to interrupt malaria transmission and probability under the T90+bed net intervention. Filled symbols indicate times to interruption. Bars illustrate the proportion of simulations in which transmission was not interrupted within 5,000 days (13.7 years). Ten simulations using identical starting conditions for each initial EIR were conducted.

Three primary interventions, plus combinations, were simulated for both baseline conditions. At the lower transmission (EIR∼3) all interventions except T90 interrupted transmission and this was sustained until the end of the simulation (Table 2). The time to reach the 150 day criterion varied between simulations of the same intervention, highlighting the stochastic nature of the process (Table 2). The T90+bed net intervention could interrupt transmission in simulations with an initial EIR<7 (Figure 2). However this was not always achieved within 13.7 years (5,000 days); the proportion of simulations resulting in interrupted transmission decreased while the time to elimination tended to increase with increasing EIR (Figure 2).

**Table 2:** Ability of simulated interventions to interrupt malaria transmission and control reintroductions.

Intervention	Low transmission (EIR ∼3)		Moderate transmission (EIR ∼15)	
	% interrupting transmission (time^a^)	% where introductions become established	% interrupting transmission (time^a^)	% where introductions become established
T90^b^	0	-	0	-
MDA^c^	100 (174–1,088)	100	40 (206–414)	100
Bed^d^	100 (1,235–4,458)	0	0	-
T90+Bed	100 (1,034–1,904)	0	0	-
MDA+Bed	100 (171–1,209)	0	65 (201–774)	100
MDA+T90	100 (174–337)	0	80 (205–679)	0
MDA+Bed+T90	100 (71–371)	0	85 (200–562)	0

a Range of time (days) until transmission is interrupted. Time to interruption is the first occurance of 150 days with no infectious mosquitoes.

b 90% chance of symptomatic individuals receiving curative treatment; time between appearance of symptoms and treatment is decreased (compared to baseline).

c 100% of population is treated with a curative dose at start of intervention and 40 days later with same drug as used for chemotherapy.

d 80% of the population receive (and use) bed nets; use of a bed net reduces mosquito exposure by 80%.

**Figure 3:**
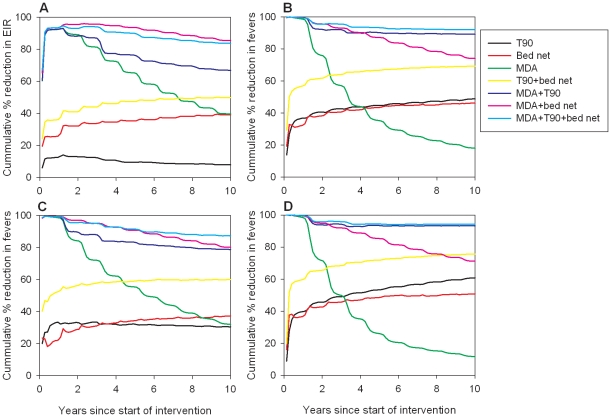
Average long-term impacts of interventions on EIR and morbidity. Impacts for (A) EIR, (B) population morbidity, (C) morbidity in 0–3 year olds and (D) >14 year olds are given relative to baseline for moderate transmission simulations. Only results from simulations in which elimination was not achieved and sustained are included, reducing the sample sizes to n = 3 and n = 5 for the MDA+bed+T90 and MDA+T90 interventions, respectively.

In the moderate transmission setting the triple combination of MDA+T90+bed nets was the most successful intervention at interrupting transmission and controlling subsequent parasite re-introduction (Table 2). Using this intervention, interrupted transmission was achieved in 85% of simulations 200–562 days after the start of the intervention. The combination of MDA+T90 was almost as effective as the triple combination (80% success in interrupting transmission) and was also able to contain new parasite introductions. Although the T90 and bed net interventions were not able to interrupt transmission, alone or in combination, both reduced the number of fever episodes, and to a lesser extent, the EIR compared to baseline (Figure 3). The relative benefits of the interventions on morbidity were found to be age specific, such that the relative reduction in fevers was larger in 0–3 year olds compared to >14 year olds for the T90, bed net, T90+bed net, MDA+T90 and MDA+T90+bed net interventions, whereas the MDA and MDA+bed net interventions had a larger relative impact in >14 year olds compared to 0–3 year olds (Figure 3).

**Figure 4:**
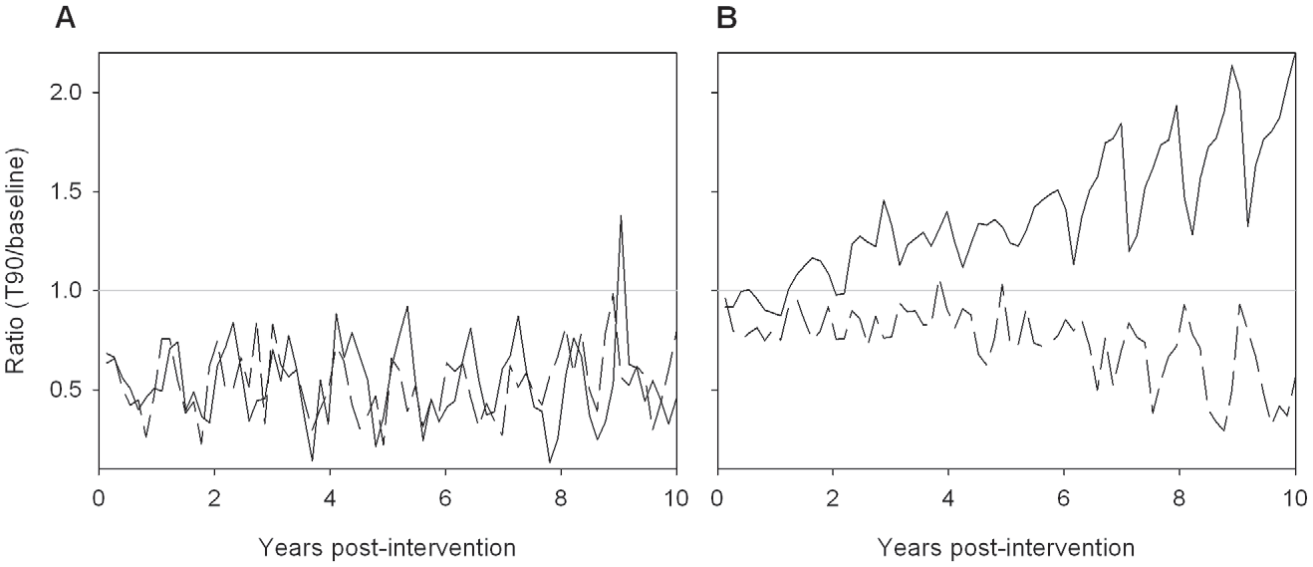
Simulated impact of T90 intervention on parasitemia. Data are expressed as the ratio of mean number of patent (≥100 parasites/µl; dashed line) or sub-patent (<100 parasites/µl; solid line) infections to baseline in (A) 0–3 year olds and (B) >14 year olds. Values <1 represent fewer infections under the intervention compared to baseline, while >1 represents more infections under the intervention.

Output from the T90 intervention in the moderate transmission setting was analysed further to investigate why the EIR consistently started to increase after 2 years of the intervention, even though morbidity continued to decline (Figure 3). This trend was observed in all simulations of this intervention. This analysis indicated that the T90 intervention caused a decrease in the proportion of people aged >14 years with patent parasitemia, but an increase in sub-patent parasite carriage relative to baseline (Figure 4). The increase in sub-patent parasite carriage only occurred in older age groups (Figure 4).

**Figure 5:**
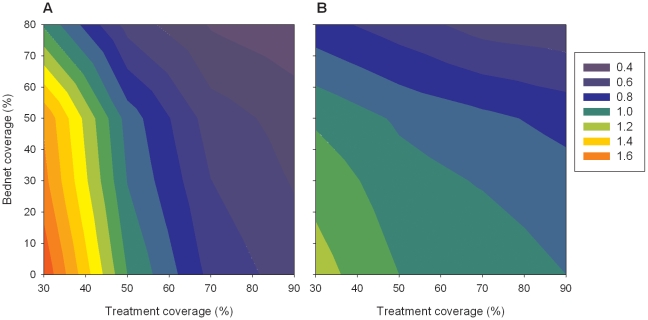
Sensitivity of model output to treatment coverage. Changes in (A) fever and (B) EIR are compared to baseline (50% treatment and 0% bed net coverage). Contours represent the ratio of intervention to baseline 5 years after start of intervention.

The sensitivity analysis indicated that the impact of the bed net and symptomatic treatment interventions was dependent on intervention coverage with higher coverage having larger impacts on transmission and morbidity (Figure 5). The relationship between changing intervention coverage and impact were not linear, particularly for morbidity where bed nets had little impact until coverage reached at least 50% (Figure 5). To assess the sensitivity of the model output to variations in the assumed efficacy of bed nets, the model was implemented with 80% coverage and efficacy of 75% or 85%. Compared to the initially assumed 80% efficacy, a reduction to 75% resulted in an average 7.1% increase in EIR, and 18.9% increase in fever. This equates to 1.19 additional fevers per person over the first 5 years of the intervention. Improving the efficacy from 80% to 85% reduced EIR and fever by an average of 12.1% and 11.8%, respectively.

## Discussion

We used a stochastic simulation model of *P. falciparum* disease and transmission to assess the impact of three types of interventions in villages with low to moderate transmission. This study focused on low to moderate transmission as countries in these regions are most likely to undertake elimination activities in the current program. Prior to the interventions, the low transmission scenario (EIR∼3) represented a community with no acquired clinical immunity and low multiplicity of infection. In contrast, the community in the moderate transmission scenario (EIR ∼15) had some acquired clinical immunity, evidenced by the decreasing frequency of fever episodes with age. The combination of seasonal transmission and a long-acting anti-malarial drug produced a relatively low number of fevers in the youngest age group, compared to the number of infections. The parasite dynamics within this community was similar to the pattern reported for a small population in Brazil [36].

Our results for the generalized communities demonstrate that interruption of *P. falciparum* transmission for at least 150 days using currently available tools is possible in both low and moderate transmission settings, supporting the results of previous studies [8], [16], [37]. As expected, the likelihood of interrupted transmission decreased with increasing EIR, while the time required increased. It is important to note that the time estimates are a best case scenario under the simulated conditions since the interventions were assumed to be deployed and active on the nominated day of implementation. This is in contrast to the usual field deployment of interventions which are rolled out over an extended period. Also it has been shown that population size impacts on the timeliness of responses to interventions, particularly when approaching elimination [15]. Further analyses are required to investigate the impact of population size on the time to interrupted transmission reported here.

Although it was possible for many of the simulated interventions to interrupt transmission, stochastic influences meant it was usually not guaranteed. The current results were achieved in an environment where parasite introductions were included, a point of difference to other models of intervention impacts [37]. It has been reported that even a small amount immigration of infected people can impact on the ability to eliminate malaria [8]. We found parasite introduction generated highly variable interruption times in the low transmission setting, but that all interventions except MDA were able to prevent parasite introductions becoming established. In the moderate transmission setting, only combination interventions involving improved treatment of symptomatic cases were able to successfully stop introductions becoming established.

Although not recommended by WHO [6], MDA has been deployed in Cambodia to demonstrate its role as a tool for malaria elimination [38]. In the current study, all interventions using MDA had an immediate and marked impact on morbidity and transmission, however this impact decreased over time in simulations where transmission was not interrupted or reintroduction was not prevented. This was particularly the case for MDA used in isolation. The relative benefits on morbidity were noted to be age-specific and this is attributable to the presence of acquired clinical immunity in older individuals. Simulation results from a different model show a similar rebound effect when regularly administered mass screening and treatment interventions cease [37]. Therefore, the short-lived benefits of MDA reinforce that it should only be used in combination with other interventions.

The model structure developed is such that interventions are directly simulated, meaning that it is not necessary to make assumptions a priori about the downstream impacts of the intervention. Instead the model's dynamic nature where transmission, host infectiousness and host immunity are intimately linked, changing over time, allows downstream intervention impacts to evolve during the simulation. To achieve these features the model is more computationally complex than many other deterministic models, and while simple models appear to replicate the major features predicted by more complex models, at least in short-term predictions [39], the results of this study highlight one of the advantages of using a more detailed model; the ability to detect unexpected or subtle changes in the infection profile within the community. Such a change occurred under the T90 intervention in the moderate transmission setting. This intervention caused the EIR to initially decrease by 14%. However after two years the EIR started to increase even though morbidity continued to decline. This increase was consistently seen in all simulations of this intervention suggesting an underlying pattern, rather than stochastic variation. Further investigation revealed that in older individuals the number of patent infections decreased compared to baseline but the number of sub-patent infections increased. This pattern, which does not occur in younger age groups, resulted from the decreased likelihood of introduced parasites becoming established in the population.

Anti-PfEMP1 antibodies provide variant-specific protection against disease [40], and this is reflected in the components of the within-host model. This means semi-immune hosts are more likely to develop symptoms against antigenically different parasites such as those newly introduced into a community. In the T90 intervention there is a high likelihood of symptomatic episodes being treated. Because newly introduced parasites are more likely to produce symptoms, they are also more likely to be terminated by chemotherapy compared to existing parasites, thereby reducing their survival probability. This bias influences the long-term parasite population structure, in favour of existing parasites, resulting in a decrease in the incidence of fever and subsequent treatment, masking the ongoing transmission of parasites in semi-immune hosts.

This observation has not been reported elsewhere since other models of malaria do not typically contain the detail necessary to monitor individual host responses to specific parasite genotypes, or track parasite genotypes. The exception is McKenzie et al [16] who introduced parasite cross-reactivity and found greater parasite persistence in the absence of parasite cross-reactivity. Our results in low to moderate transmission settings show a similar result in the absence of treatment, but when treatment of symptomatic individuals is included the higher likelihood of “new” parasites to generate symptoms and thus be terminated by chemotherapy before transmission outweighs the with-in host parasite selection caused by host immunity.

It is common for the impact of control and elimination programs to be monitored using data on malaria morbidity and mortality and slide positive rates, particularly in children [6]. However our results predict that changes in morbidity over-estimate the corresponding changes in transmission, and that the impacts on morbidity are often age-specific. As expected, the impacts of the simulated interventions are sensitive to intervention coverage, although this relationship is not linear. The model output is also likely to be sensitive to the assumed number of feeding attempts made by a mosquito, although this parameter did not form part of the sensitivity analysis. Under the assumed 3 feeding attempts, the sensitivity analysis revealed the model output, particularly the prevalence of fever, is sensitive to the assumed efficacy of the bed net intervention. Considering fever, a bed net intervention with efficacy of 75% and 80% coverage had the same impact as a bed net intervention with 80% efficacy and ∼68% coverage. Bed net efficacy depends on a number of factors, some of which are able to be influenced (eg education on proper use, regular maintenance and repair) and some which are inherent to a region (eg feeding characteristics of mosquito population), providing a source of variability when comparing the impact of the intervention between regions which appear to have the same overall net coverage. In this generalized model we have only considered the effect of untreated nets. However similar conclusions have been reported for ITN interventions [41], [42], with ITN effectiveness being sensitive to the assumed killing and repellancy effect of the insecticide, in addition to coverage [37].

This study quantifies the results from a new stochastic simulation model applied to villages undergoing low to moderate transmission. The results add further information to the public domain, information ultimately aimed to help policy-makers and public health officials better plan and combat malaria. The results also highlight the potential under some interventions for a shift towards sub-patent parasite carriage which would often not be detected in either case data or standard malaria surveillance. This model outcome warrants further investigation in the field because if confirmed, this feature could impact on the success of elimination efforts even in regions of low to moderate transmission.
